# EXAMINING DEMENTIA AND NONDEMENTIA CAREGIVERS’ NUTRITION AND OVERALL HEALTH

**DOI:** 10.1093/geroni/igad104.3315

**Published:** 2023-12-21

**Authors:** Abigail Nehrkorn-Bailey, Tanner Edmonds, Alissondra Quatsoe

**Affiliations:** University of Wisconsin-Green Bay, Green Bay, Wisconsin, United States; University of Wisconsin-Green Bay, Green Bay, Wisconsin, United States; University of Wisconsin-Green Bay, Green Bay, Wisconsin, United States

## Abstract

The literature on dementia caregivers is sparse. A study from Sheehan et al. (2021) concluded that the well-being of dementia caregivers and nondementia caregivers largely did not differ. To note, the sample size in that study was limited and no health behaviors were assessed. This study extends and adds to the Sheehan et al. (2021) study by utilizing the July 2023 Behavioral Risk Factor Surveillance System data (CDC, 2023) to examine nutrition (i.e., fruit and vegetable intake) and overall physical and mental health among (1) dementia caregivers and (2) nondementia caregivers. Two multiple mediation analyses were conducted comparing caregiving group (n = 4,853 dementia caregivers; n = 28,698 nondementia caregivers): physical health was the outcome in Model 1, and mental health was the outcome in Model 2. The direct pathway from caregiving group to (1) physical health in Model 1 was significant (b = -.05, p < .001) and (2) mental health in Model 2 was significant (b = -0.11, p < .001). The pathway from caregiving group to vegetable intake was significant in both models, but the caregiving group-fruit intake pathway was not significant. The pathways from vegetable and fruit intake to physical and mental health were all significant. Lastly, vegetable intake significantly mediated the pathway from caregiving group to (1) physical health (b = 0.001, CI = [.0003 to .0020]) and (2) mental health (b = 0.001, CI = [.0002 to .0015]). These findings indicate important diet and overall health differences between dementia and nondementia caregivers.

